# Literature Retrieval and Mining in Bioinformatics: State of the Art and Challenges

**DOI:** 10.1155/2012/573846

**Published:** 2012-06-21

**Authors:** Andrea Manconi, Eloisa Vargiu, Giuliano Armano, Luciano Milanesi

**Affiliations:** ^1^Institute for Biomedical Technologies, National Research Council, Via F.lli Cervi, 93, 20090 Segrate, Italy; ^2^Department of Electrical and Electronic Engineering, University of Cagliari, Piazza d'Armi, 09123 Cagliari, Italy; ^3^Barcelona Digital Technological Center, C/Roc Boronat 117, 08018 Barcelona, Spain

## Abstract

The world has widely changed in terms of communicating, acquiring, and storing information. Hundreds of millions of people are involved in information retrieval tasks on a daily basis, in particular while using a Web search engine or searching their e-mail, making such field the dominant form of information access, overtaking traditional database-style searching. How to handle this huge amount of information has now become a challenging issue. In this paper, after recalling the main topics concerning information retrieval, we present a survey on the main works on literature retrieval and mining in bioinformatics. While claiming that information retrieval approaches are useful in bioinformatics tasks, we discuss some challenges aimed at showing the effectiveness of these approaches applied therein.

## 1. Introduction

Nowadays, most of the scientific publications are electronically available on the Web, making the problem of retrieving and mining documents and data a challenging task. To this end, automated document management systems have gained a main role in the field of intelligent information access [1]. Thus, research and development in the area of bioinformatics literature retrieval and mining is aimed at providing intelligent and personalized services to biologists and bioinformaticians while searching for useful information in scientific publications. In particular, the main goal of bioinformatics text analysis is to provide access to unstructured knowledge by improving searches, providing automatically generated summaries, linking publications with structured resources, visualizing contents for better understanding, and guiding researchers to formulate novel hypotheses and to discover knowledge.

In the literature, several methods, systems, and tools to retrieve and mine bioinformatics publications have been proposed and adopted, some of them being currently available on the Web. In this paper, we provide a survey of existing end-user-oriented literature retrieval and/or mining solutions for bioinformatics, together with a short discussion on open challenges. The rest of the paper is organized as follows: Section 2 illustrates the main topics addressed in this paper, that is, information retrieval, text mining, and literature retrieval and mining. In Section 3, the state of the art on literature retrieval and mining in bioinformatics is presented.  Section 4 discusses some relevant open problems and challenges.  Section 5 ends the paper.

## 2. Background

Supporting users in handling the huge and widespread amount of Web information is becoming a primary issue. Information retrieval is the task of representing, storing, organizing, and accessing information items. Information retrieval has considerably changed in recent years: initially with the expansion of the World Wide Web and the advent of modern and inexpensive graphical user interfaces and mass storage [2], and then with the advent of modern Internet technologies [3] and of the Web 2.0 [4].

Information retrieval can cover various and heterogeneous kinds of data and information problems beyond that specified in the core definition above. More generally, an information retrieval system does not inform (i.e., does not change the knowledge of) the user on the subject of her/his inquiry. It merely informs on the existence (or nonexistence) and whereabouts of documents relating to her/his request. According to [5], information retrieval is defined as the task of finding material (usually documents) of an unstructured nature (usually text) that satisfies an information need, from large collections (usually stored on computers). Nowadays, information retrieval solutions rely on the adoption of Web services and suitable Semantic Web approaches, such as ontologies. Indeed, Semantic Web inference can improve traditional text search, and text search can be used to facilitate or augment Semantic Web inference [6].

Text Mining is an information retrieval task aimed at discovering new, previously unknown information, by automatically extracting it from different text resources [7]. In fact, the term “text mining” is generally used to denote any system that analyzes large quantities of natural language text and detects lexical or linguistic usage patterns in an attempt to extract probably useful (although only probably correct) information [8]. Automatic extraction of metadata (e.g., subjects, language, authors, key-phrases) is a prime application of text mining techniques. Although contemporary automatic document retrieval techniques bypass the metadata creation stage and work directly on the full-text of the documents, text mining has been largely applied to learn metadata from documents. Language identification is a relatively simple mining task aimed at providing an important piece of metadata for documents in international collections. A simple representation for document categorization is to characterize each document by a profile that consists of “*n*-grams,” that is, sequences of *n* consecutive words, that appear in it. Occurrence probabilities of common words are then compared with the most frequent words of the text data. Author's metadata is one of the primary attributes of most documents, and it is usually known. However, in some cases, authorship is uncertain and must be guessed from the text. Text mining is also applied to provide summaries of documents or groups of documents. Text summarization is aimed at producing a condensed representation of its input, intended for human consumption [9]. Earliest instances of research on summarization of scientific documents extract salient sentences from text using features like word and phrase frequency [10], positions in the text [11], and key phrases [12]. Various works published since then had concentrated on other domains, mostly on newswire data [13] and contextual advertising [14]. Overall, summarization techniques can be divided in two groups [15]: those that extract text containing the most relevant information from the source documents (*extraction-based approaches*) and those that perform paraphrasing on the source documents (*abstraction-based approaches*).

Document clustering is an unsupervised learning technique in which there is no predefined category or class, but groups of documents that belong together are sought. For example, document clustering may assist in retrieval tasks by creating links between similar documents, which in turn allows related documents to be retrieved once one of the documents has been deemed relevant to a query [16]. Although they do not require training data to be preclassified, clustering techniques are generally often more computation intensive than supervised schemes [17]. Nevertheless, clustering has been largely applied in text mining applications. Trials of unsupervised schemes include the work by Aone et al. [18], who use the conceptual clustering scheme COBWEB [19] to induce natural groupings of close-captioned text associated with video newsfeeds; Liere and Tadepalli [20], who explore the effectiveness of AutoClass [21] in producing a classification model for a portion of the Reuters corpus; Green and Edwards [22], who use AutoClass to cluster news items gathered from several sources into *stories* (i.e., groupings of documents covering similar topics). One of the main subfields of text mining is information extraction, that is, the task of filling templates from natural language input [23]. Typical extraction problems address simple relationships among entities, such as finding the predicate structure of a small set of predetermined propositions. Machine learning has been applied to the information extraction task by seeking pattern-match rules that extract fillers for slots in the template [24–27]. Extracted information can be used in a subsequent step to learn rules that characterize the content of the text itself.

In the academic area, online search engines are used to find out scientific resources, as journals and conference proceedings. However, finding and selecting appropriate information on the Web is still difficult. To simplify this process, several frameworks and systems have been developed to retrieve scientific publications from the Web. Bollacker et al. [28] developed CiteSeer (http://citeseer.ist.psu.edu/), the well-known automatic generator of digital libraries of scientific literature. Being aimed at eliminating most of the manual effort of finding useful publications on the Web, CiteSeer uses sophisticated acquisition, parsing, and presentation methods. In particular, CiteSeer uses a three-stage process: database creation and feature extraction; personalized filtering of new publications; personalized adaptation and discovery of interesting research and trends. These functions are interdependent: information filtering affects what is discovered, whereas useful discoveries tune the information filtering. In [29], the authors study how to recommend research publications using the citation between publications to create a user-item matrix. In particular, they test the ability of collaborative filtering to recommend citations that could be additional references for a target research publication. Janssen and Popat [30] developed UpLib, a personal digital library system that consists of a full-text indexed repository accessed through an active agent via a Web interface. UpLib is mainly concerned with the task of collecting personal collections comprising tens of thousands of documents. In [31], Mahdavi et al. start from the assumption that trend detection in scientific publication retrieval systems helps scholars to find relevant, new and popular special areas. To this end, they developed a semiautomatic system based on a semantic approach.

## 3. State of the Art

A great deal of biological information accumulated through years is currently available in online text repositories such as Medline. These resources are essential for biomedical researchers in their everyday activities to plan and perform experiments and verify the results.

Among other kinds of information, let us concentrate on publications and scientific literature, largely available on the Web for any topic. As for bioinformatics, the steady work of researchers, in conjunction with the advances in technology (e.g., high-throughput technologies), has arisen in a growing amount of known sequences. The information related with these sequences is daily made available as scientific publications. Digital archives like BMC Bioinformatics (http://www.biomedcentral.com/bmcbioinformatics/), PubMed Central (http://www.pubmedcentral.gov/) and other online journals and resources are more and more searched for by bioinformaticians and biologists, with the goal of retrieving publications relevant to their scientific interests. For researchers, it is still very hard to find out which publications are of interest without an explicit classification of the relevant topics they describe. Thus, these resources must provide adequate mechanisms for retrieving the required information.

### 3.1. Literature Retrieval in Bioinformatics

Discovering and accessing the appropriate bioinformatics resource for a specific research task has become increasingly important, as suggested in earlier reports [32]. To address this issue, various significant projects and initiatives have been carried out, leading to several pioneering indexes of bioinformatics resources that are currently available on the Internet. Available search engines can be categorized according to different criteria. In particular, in agreement with [33], search engines can be categorized in three groups, depending on the way a query is performed: (i) those that perform the query only in the fields of citations; (ii) those that perform the query in the full text article; (iii) those that further process the retrieved citations to organize them and/or to retrieve further information.

As for the first category, let us recall here RefMed [34], MedlineRanker [35], and iPubMed [36]. RefMed (http://dm.postech.ac.kr/refmed/) is a search engine for PubMed that provides relevance ranking. It is widely known that ranking according to the global importance often does not meet the user interests. Given a starting keyword-based query, an initial list of results is presented to the user, who analyzes the proposed documents and passes judgment on their relevance. Then, RefMed induces a new ranking according to the user judgment by exploiting a machine learning algorithm (i.e., RankSVM [37]). MedlineRanker (http://cbdm.mdc-berlin.de/tools/medlineranker/) and iPubMed (http://ipubmed.ics.uci.edu/) are search engines for Medline. For a given topic, the former learns the most discriminative words by comparing a set of abstracts provided by the user with the whole Medline (or a subset). Then, it ranks abstracts according to the learned discriminative words. The latter, which implements the *search-as-you-type* paradigm, has the main advantage to provide results on the fly, which allows users to dynamically modify their query.

eTBLAST [38] and QUERTLE [39] belong to the second category. eTBLAST (http://etest.vbi.vt.edu/etblast3/) allows searching for both citations (i.e., Medline) and full-text articles (i.e., PubMed Central). To retrieve useful documents, it performs a text-similarity search by comparing documents in a target database with an input text. In doing so, it finds the documents that best match the keywords extracted from the query by analyzing the word alignment.

QUERTLE (http://www.quertle.info/) is a new semantic search engine able to perform queries on PubMed, Toxline, National Institutes of Health Re-PORTER, PubMed Central and BioMed Central. Unlike the above-mentioned systems, QUERTLE is able to perform queries based on the meaning and the context of documents. It exploits a meta-database of subject-verb-object relationships asserted by the authors and automatically extracted using semantic-based linguistics. The search engine matches the user query against these relationships.

Finally, GoPubMed [40], XploreMed [41], EBIMed [42], and iHOP [43] are search engines that belong to the third category. GoPubMed allows (http://www.gopubmed.com/web/gopubmed/) submitting keywords to PubMed, extracts Gene Ontology (GO) terms from the retrieved abstracts (GO is becoming a standard for gene/protein function annotation), and supplies the user with the relevant ontology for browsing. It indexes PubMed search results with ontological background knowledge, such as GO and MeSH. The approach to search can also help to answer questions. In particular, the summary of important terms in “top five & more” is a most helpful feature for answering questions or reducing the big initial result to a smaller set of relevant publications in one click. XploreMed (http://www.ogic.ca/projects/xplormed/) filters PubMed results according to the eight main MeSH (http://www.nlm.nih.gov/mesh/) categories and then extracts topic keywords and their cooccurrences, with the goal of extracting abstracts. EBIMed (http://www.ebi.ac.uk/Rebholz-srv/ebimed/) combines information retrieval and extraction from Medline. It analyzes retrieved Medline abstracts to highlight associations among UniProtKB/Swiss-Prot proteins, GO terms, drugs, and species. All identified terms, sentences, and abstracts are displayed in tables, and all terms are linked to their entries in biomedical databases. iHOP (http://www.ihop-net.org/UniPub/iHOP/) uses genes and proteins as hyperlinks among sentences and abstracts. It converts the information in PubMed into navigable resources. The navigation along gene network allows a stepwise and controlled exploration of the information space. Each step through the network produces information about one single gene and its interactions.

### 3.2. Literature Mining in Bioinformatics

 Given the growth of biomedical information on the Internet, Web-based tools capable of mining the public databases and of highlighting their relevant information in a well-organized and coherent manner are more and more required. Tanabe et al. [44] have proposed MedMiner (MedMiner is no longer available), an Internet-based hypertext program able to filter and organize large amounts of textual and structured information returned from public search engines—like GeneCards (http://www.genecards.org/) and PubMed. MedMiner offered a potentially significant new aid for coping with the torrent of molecular biology data confronting today researchers. By filtering and organizing material retrieved from high-quality Internet sites, it makes complex database searches much easier to execute and digest. MedMiner successfully integrated public and local databases, using a local database as a “proxy” to the (much larger) public ones. Additional databases could be merged into the system, integrating a wider variety of filters with a consistent user interface. PubCrawler (http://pubcrawler.gen.tcd.ie/) is a free alerting service that scores daily updates to the NCBI Medline [45] and GenBank databases. PubCrawler can keep scientists informed of the current contents of Medline and GenBank by listing new database entries that match their research interests.

To facilitate retrieval and analysis of the huge amount of data contained in documents on biological and medical data, several researchers developed dedicated information extraction systems that attempt to simplify the underlying tasks. Most of the corresponding works use the abstract only, owing to the convenience of access and the quality of data.

As abstracts provide a concise summarization of a publication, very useful to categorize it. On the other hand, analyzing full text is essential to detect all detailed information (e.g., methods, tables, and figures). Abstracts are generally available through central collections with easy direct access (e.g., PubMed). Full texts are distributed across many locations (e.g., publishers websites, journal websites, and local repositories), making their access more difficult [46].

Interactions between proteins are very important to understand their functions and biological processes. Several approaches and tools have been defined to deal with this challenge. Thomas et al. [47] present a system aimed at extracting occurrences of protein interactions from Medline abstracts, producing a database of protein pairs characterized by a type of interaction. To this end, the authors customized the Highlight system, a general purpose information extraction engine for commercial applications [47]. The main customizations of highlight consist of (i) adapting the natural language component to make it able to correctly recognize the relevant entities and events (ii) developing a set of templates or outlines of the kinds of relevant information, and (iii) developing patterns aimed at deciding how to slot items and events into templates. PPI Finder (liweilab.genetics.ac.cn/tm/) [48] is a web application aimed at mining human protein-protein interactions from PubMed abstracts. It is able to (i) find the genes related to the gene of interest based on their cooccurrence frequencies and (ii) extract the semantic descriptions of interactions from the co-occurring literature by computational linguistic methods. Moreover, PPI Finder maps the known interactions from the widely used PPI databases, with the aim to distinguish between novel and known interactions. PIE (http://pie.snu.ac.kr/) (Protein Interaction information Extraction) is a web application to extract protein-protein interaction sentences from PubMed abstracts as well as user-provided articles. To extract hidden interactions, PIE exploits natural language processing and machine learning techniques.

Another important challenge is to automatically translate biomedical literature text into a structured form. Due the huge increase of biomedical literature, manual annotation databases are often incomplete and inconsistent with the literature [49]. In this perspective, Craven and Kumlien [50] applied machine learning techniques to automatically map information from text sources to structured representations. In particular, the goal of their research is to develop methods that can accurately map information from scientific text sources to structured representations, such as knowledge bases or databases. To this end, they developed a system to automatically extract key facts from scientific texts. Their system could be used as a support to construct and update databases and knowledge bases. The authors used the system in the development of an ontology of localization patterns and to populate the corresponding database with text-extracted facts describing localization patterns of individual proteins. Another application of this system is to provide structured summaries of what is known about biological objects. Moreover, according to Swanson and Smalheiser [51], the system can be used to extract relationships among entities by automatically eliciting information from the literature. PreBIND (http://bind.ca) [52] is a system developed to solve a very specific problem. It has been devised to curate the BIND database. BIND is a database aimed at curating and archiving protein-protein interaction from the literature using a standard data representation. In doing so, PreBind exploits both statistical and rule-based methods. Statistical methods are used to retrieve relevant documents, whereas rule-based methods are used for biomolecule name recognition, with the aim to find statements about protein interactions. Wiegers et al. [53] proposed another tailored solutions. The authors presented a text-mining prototype to curate the Comparative Toxicogenomics Database (CTD), a publicly available resource that promotes understanding about the etiology of environmental diseases. It provides manually curated chemical-gene/protein interactions and chemical- and gene-disease relationships from the peer-reviewed published literature. The goals of the research reported here were to establish a baseline analysis of current CTD curation, develop a text-mining prototype from readily available open-source components, and evaluate its potential value in augmenting curation efficiency and increasing data coverage. PathText [54] is an integrated environment for combining standards compliant biological pathway models and original publications regarding selected parts of the pathway, through the use of text mining technology and tools, to facilitate the creation of manual annotations. PathText integrates three knowledge sources indispensable for systems biology: (i) external databases, such as SwissProt, EntreGene, Flybase, HUGO; (ii) text databases such as MEDLINE and full publications; (iii) pathways as organized interpretations of biological facts. PathText successfully provides integration of text to pathways and has been used by three groups that make research on biological topics at the Systems Biology Institute, the University of Tokyo [55], and the Manchester Centre for Integrative Systems Biology in the UK [56]. Karamanis et al. [57] apply natural language processing techniques to develop a generic tool aimed at assisting FlyBase curators. Kiritchenko et al. [58] proposed a tool aimed at retrieving Medline publications that mention genes. After being retrieved, publications are categorized according to the GO codes. The purpose of their work is to retrieve the known functionality of a group of genes from the literature and translate it into a controlled vocabulary. The categorization process can be used for automatic or semiautomatic database curation and maintenance. At the same time, it can be used as a stage in gene expression analysis. After that microarray experiments have been performed and gene expression data have been preprocessed and clustered, the information on gene functions can be added as background knowledge. Literature-Based Discovery (LBD for short) is another relevant research area that applies text-mining with the goal of finding new relationships from knowledge typically available on the Web, in terms of scientific documents, books, and papers. The technique was pioneered by Don R. Swanson in the 1980s and has been widely studied afterwards. It is worth pointing out that LBD techniques do not generate knowledge by means of experiments. Rather, they seek to connect existing knowledge from empirical results by searching and highlighting relationships not yet put into evidence. The pioneering work of Swanson [59] hypothesized the role of fish oil in clinical treatment of Raynaud's disease, combining different pieces of information from the literature, and the hypothesis was later confirmed with experimental evidence. Swanson was using the so-called ABC model of discovery, which asserts that, in the event A and B are related and B and C are related, then A and C might be (indirectly) related. Swanson's ABC model can be implemented in accordance with two different discovery processes: closed and open. The former tries to identify existing links between a hypothesis and the existing literature, whereas the latter generalizes the closed approach by rendering the hypothesis a “free variable” in the discovering task. Hence, a closed discovery process is characterized by the elaboration of a hypothesis, whereas an open discovery process is also concerned with hypothesis generation. LBD has been extensively investigated and applied to many areas of biomedicine, mainly using textual information derived from MedLine (typically in terms of titles, abstracts, and MeSH headings). Among relevant tools and systems proposed and/or experimented in this research field (for a review, see, e.g., [60]), let us recall the work of Hristovski et al. [61]. The authors use semantic predications to enhance cooccurrence-based open discovery systems. Predications are produced by using two natural language processing systems in combination that is, BioMedLEE [62] and SemRep [63], together with the BITOLA system. BITOLA is an open discovery system, compliant with the Swanson's approach, which uses MeSH terms instead of title words and employs association rules instead of word frequencies to relate medical concepts. The authors include also domain-specific knowledge, as they use information in the form of chromosome location and gene expression localization.

Corney et al. [64] propose BioRAT (http://bioinf.cs.ucl.ac.uk/software_downloads/biorat/) (Biological Research Assistant for Text mining), an information extraction tool specifically tailored for biomedical tasks. Able to access and analyze both abstracts and full-length publications, it incorporates a domain specific document search ability. BioRAT uses natural language processing techniques and domain-specific knowledge to search for patterns in documents, with the aim of identifying interesting facts. These facts can then be extracted to produce a database of information, which has a higher *information density* than a pile of publications. PolySearch (http://wishart.biology.ualberta.ca/polysearch/) [65] is a web application aimed at extracting and analyzing text-derived relationships between human diseases, genes, proteins, mutations (SNPs), drugs, metabolites, pathways, tissues, organs, and subcellular localizations. To this end, it analyzes documents and data from several sources, including PubMed, OMIM, DrugBank, SwissProt, HMDB, HGMD, and Entrez SNP. The system has been designed to address queries of the form “*Given a single X, find all Y's*,” where X and Y are biomedical terms (e.g., diseases, tissues, cell compartments, and gene/protein names). Metabolic and signaling pathways are an increasingly important part of organizing knowledge in systems biology. They serve to integrate collective interpretations of facts scattered throughout the literature. Biologists construct a pathway by reading a large number of publications and interpret them as a consistent network, but most of the models currently lack direct links to those publications. Biologists who want to check the original publications have to spend substantial amounts of time to collect relevant publications and to identify the sections relevant to the pathway [66]. PathwayAssist [67] is a software application developed to navigate and analyze biological pathways, gene regulation networks, and protein interaction maps. PathwayAssist enables researchers to create their own pathways and produces pathway diagrams. For visualization purposes, pathways are represented as a graph with two types of nodes: those reserved for proteins, small molecules, and cellular processes and those that represent events of functional regulation, chemical reactions, and protein-protein interactions. PathwayAssist comes with a database of molecular networks automatically assembled from scientific abstracts. The database has been populated by using the text-mining tool MedScan on the whole PubMed. MedScan preprocesses input text to extract relevant sentences, which undergo natural language processing. The preprocessing step uses a manually curated dictionary of synonyms to recognize biological terms. Sentences that do not contain at least one matched term are filtered out. The natural language processing kernel deduces the syntactic structure of a sentence and establishes logical relationships between concepts. Finally, results are matched against the functional ontology to produce the biological interpretation. SciMiner (http://jdrf.neurology.med.umich.edu/SciMiner/) [68] is a web-based literature mining and functional analysis tool aimed at analyzing Medline abstracts and full-text articles to identify genes and proteins. Gene and proteins are extracted and ranked by the number of documents in which they appear. Moreover, they are further analyzed for their enrichments in GO terms, pathways, Medical Subject Heading (MeSH) terms, and protein-protein interaction networks based on external annotation resources. Anni 2.0 (http://biosemantics.org/anni) [69] retrieves documents and associations for several classes of biomedical concepts. It exploits an ontology-based interface to MEDLINE that defines concepts and their relations. Anni finds related concepts based on their associated sets of texts. Peregrine [70] is a concept recognition software, that has been used in Anni to identify references to concepts in text. Texts can be also related to a concept by using manually curated annotation databases. Texts related with a concept are characterized by a concept profile, which consists of a list of concepts used to infer functional associations between genes, between genes and GO codes, to infer novel genes associated with the nucleolus, and to identify new uses for drugs and other substances in the treatment of diseases. FACTA (http://text0.mib.man.ac.uk/software/facta/) [71] is a text search engine aimed at browsing biomedical concepts that are potentially relevant to a query. FACTA differs from other similar tools for its ability to deliver real-time responses and to accept flexible queries.

## 4. Open Problems and Challenges

As already pointed out, the steady work of researchers has brought a huge increase of publications in life sciences. This amount of scientific literature requires an extra work by researchers, typically involved in keeping up-to-date all information related to their favorite research topics. This effort mainly depends on two aspects: the continuous increase of the scientific production and the poor amount of communication among life science disciplines [72]. In this scenario, devising suitable strategies, techniques, and tools aimed at supporting researchers in the task of automatically retrieving relevant information on the Web (in particular, from text documents), has become an issue of paramount importance.

The research field of literature retrieval and mining in bioinformatics is intrinsically manifold, which makes more complex the task of identifying open problems and challenges. However, in our view, some specific issues deserve the attention of researchers more than others, along the way that leads to significant improvements. Without claiming exhaustiveness, let us briefly point out some of them: (i) encoding/preprocessing techniques; (ii) intrinsic complexity of literature retrieval and mining problems; (iii) standards and requirement for further standardization; (iv) assessment of existing tools.

**Encoding/Preprocessing Techniques**

Roughly speaking, preprocessing techniques can be divided according to the following dimensions: (i) natural language processing (NLP), (ii) lexical techniques, and (iii) semantic techniques. Currently, NLP does not guarantee to come up with effective solutions able to account for the virtually infinite set of variations concerning the way relevant information is “deployed” in text documents. However, this field may become of primary importance in the next future, due to its great potential. Lexical techniques, focused on finding relevant terms able to characterize documents, are usually simpler to implement, no matter whether they are actually framed in a perspective based on frequencies or information theory. As a matter of fact, they should be considered only a starting point, as preprocessing made using purely lexical techniques (e.g., TFIDF [2]) appears not suitable for typical literature retrieval and mining problems. To some extent, semantic techniques lie in the middle between NLP and lexical techniques. A usual schema adopted while applying semantic techniques is to enrich lexical information with additional knowledge, which can be obtained in several ways. Just to cite few: (i) any given text document can be mapped to an existing taxonomy/ontology, with the goal of identifying relevant concepts and attach them to the document itself, to facilitate further processing; (ii) specific term disambiguation techniques (e.g., latent semantic indexing, synset analysis, or NER analysis) may be applied, with the goal of improving the significance of candidate terms, to be used for representing (together with other terms) a given document; (iii) space transformation techniques (e.g., feature extraction) may be applied, with the goal of limiting the amount of information required for disambiguating text documents. Based on singular value decomposition, Latent Semantic Indexing (LSI) [73] is a technique used to compute document and term similarities according to a “soft” term matching rule. In doing so, terms and documents can be expressed as vectors of statistically independent factors, so that the similarity of any two terms can be better estimated by the cosine of their vector expressions. Synsets have become popular with the advent of WordNet [74], a lexical database for the English language. In Wordnet, English words are grouped into sets of synonyms called synsets, each containing all synonyms related to a specific concept. Named Entity Recognition (NER) [75] is aimed at detecting all instances of a biological term (e.g., protein name, gene name, drug name) in an article or a in a collection.

**Intrinsic Complexity of Literature Retrieval and Mining Problems**

Beyond the difficulties related to the task of identifying the “right” encoding/preprocessing technique to be adopted, some tasks are in fact inherently complex. For instance, let us consider a generic open discovery processes, framed in the subfield of LBD, which requires to select the hypothesis to be investigated. Even under the assumption that the corresponding task is guided by suitable heuristics aimed at restricting the set of candidate hypotheses, the complexity in time of an open discovery process remains very high and requires specific AI techniques and algorithms. Besides, at least in principle, complexity issues hold also for closed discovery processes, as they can be framed in the general context of abduction.

**Standards and Requirements for Further Standardization**

Life sciences are evolving very quickly. To this end, a wide agreement by the scientific community on describing biological concepts is more and more required. On one hand, resolving names, abbreviations, and acronyms is very difficult, due to the fact that different entities could be referenced through the same (or similar) names, abbreviations, and acronyms. On the other hand, it is difficult to detect when a composite name begins and ends in a text. In our opinion, these problems strictly depend on the lack of standard nomenclature and software tools. Fortunately, a good initiative aimed at promoting standardization has been the Unified Medical Language System (UMLS), which brings together many health and biomedical vocabularies and standards—with the goal of enabling interoperability between computer systems. The UMLS has three tools: Metathesaurus (which contains terms and codes from many vocabularies), Semantic Network (able to navigate throughout relevant categories and their relationships), and Specialist (equipped with language processing tools). However, several other problems are still open—due to a lack of standardization. In our opinion, one of the most challenging problems is the need for automatically fusing literature and biological databases. Indeed, the activities of bioinformaticians are unrelated from those of database curators. In this scenario, standard tools able to facilitate the tasks of extracting text and relationships from the literature and to facilitate database curators in the task of identifying relevant literature for annotation would greatly contribute to make the problem less severe or even absent. Other challenging problems are strongly related to the structure of scientific publications. Indeed, although it is quite easy to detect relationships between sentences by analyzing an abstract, the same it is not true while analyzing a full-text publication. This happens because the ability of a software system to detect relationships within a publication is closely related to the structure therein. In particular, each section may be in charge of addressing a specific topic. For instance, the *Introduction* is devoted to describe and analyze the problem; the *Methods* section is aimed at illustrating and explaining the methodological approach, whereas *Results* and *Discussion* are devoted to report experimental results and to discuss whether the initial goals have been achieved. This implies that different concepts (e.g., entity names, experimental conditions, and results) might be located at different sections of a publication [76]. As a consequence, a term could be related to different concepts, depending of the section(s) in which it appears. For example, the name of a gene in the *Introduction* can be related to results published in previous works rather than to novel discoveries presented in the document under analysis. The same might happen for sentences belonging to different sections of the same publication. To solve these problems, recent advances in literature retrieval and mining, together with the increase of open-access journals, are propelling publishers to provide structured version of full-text publications (usually as XML files). We completely agree that the adoption of suitable standards able to represent documents in a structured way would greatly improve the effectiveness of text mining procedures.

**Assessment of Existing Tools**

Nowadays, the scientific community is strongly concerned with finding how the proposed techniques can provide better access to the existing literature. Some competitions have been organized with the aim of assessing to which extent new approaches allow to navigate and mine the literature. A good example in this direction is given by the critical assessment of text mining methods in molecular biology (BioCreAtIvE) [77, 78]. This competition, which gets together every two years many researchers, is aimed at comparing methods devised to detect: (i) biologically significant entities and their association to existing database entries and (ii) entity-fact associations (e.g., protein-functional term associations). In our view, further initiatives in this direction could promote the sharing of relevant knowledge and skills, while pushing researchers to make a step forward in their specific topics of interest.

## 5. Conclusions

Research and development in the analysis of bioinformatics literature aims to provide bioinformaticians with effective means to access and exploit the knowledge contained in scientific publications. Although the majority of scientific publications are nowadays electronically available, keeping up to date with recent findings remains a tedious task hampered by the difficulty of accessing the relevant literature. Bioinformatics text analysis aims to improve the access to unstructured knowledge by alleviating searches, providing auto-generated summaries, linking publications with structured resources, visualizing content for better understanding, and supporting researchers in the task of formulating novel hypotheses and of discovering knowledge. Research over recent years has improved fundamental methods in bioinformatics text mining, ranging from document retrieval to the extraction of relationships. Consequently, more and more integrative literature analysis tools have been put forward, targeting a broad audience of life scientists. In this paper, after briefly introducing information retrieval, text mining, and literature retrieval and mining, we first recalled the state of the art on literature retrieval and mining in bioinformatics. In the second part of the paper, we discussed some challenges deemed worth of further investigation, with the goal of improving bioinformatics literature-retrieval-and-mining tools and systems. Summarizing, the scientific community is strongly involved in addressing different problems in literature retrieving and mining, and several solutions have been currently proposed and adopted. Nevertheless, they will remain largely ineffective until the scientific community will make further significant steps towards common standards concerning the way existing knowledge is published and shared among researchers—with particular emphasis on the structure of the scientific publications.
